# A study on the association between sleep quality and mental health of township healthcare workers from the perspective of social support

**DOI:** 10.3389/fpubh.2026.1797424

**Published:** 2026-04-10

**Authors:** Sheng Li, Yali Feng, Qing Jia, Fei Wang, Guoai Yao, Jinyu Wang

**Affiliations:** 1 The No. 2 People’s Hospital of Lanzhou, Lanzhou, China; 2 The Second Hospital of Lanzhou University, Lanzhou, China; 3 LanZhou Pulmonary Hospital, Lanzhou, China; 4 School of Public Health, Gansu University of Chinese Medicine, Lanzhou, China; 5 School of Public Health, Lanzhou University, Lanzhou, China

**Keywords:** healthcare workers, mental health, sleep quality, social support, township healthcare workers

## Abstract

**Objective:**

To explore the relationship between sleep quality and mental health among medical staff in township health centers.

**Methods:**

From September 1 to September 20, 2024, stratified random cluster sampling was used to select healthcare workers from township health centers in five districts and three counties of Lanzhou City. The Pittsburgh Sleep Quality Index (PSQI), Symptom Checklist-90 (SCL-90), and Social Support Rating Scale (SSRS) were employed to measure sleep quality, mental health, and social support levels. Partial correlation analysis was conducted to examine the relationships among these factors.

**Results:**

(1) The majority of participants were female, of Han ethnicity, and nurses. Educational attainment was primarily at the junior college and bachelor’s degree levels, with working years concentrated in the middle-aged and young backbone stage, showing a trend of high in the middle and low at both ends. The detection rate of sleep disorders was 55.70%, the SCL-90 positive rate was 45.50%, and the level of social support was moderate. (2) The total PSQI score was positively correlated with the total SCL-90 score (*r* = 0.602, *p* < 0.001) and negatively correlated with the total SSRS score (*r* = −0.272, *p* < 0.001). The total SSRS score was negatively correlated with the total SCL-90 score (*r* = −0.305, *p* < 0.001). (3) Sleep quality had a significant direct effect on mental health (direct effect proportion: 92.64, 95% CI: 6.194–8.722). Social support showed a significant partial mediating effect between sleep quality and mental health (indirect effect proportion: 7.36, 95% CI: 1.176–1.323).

**Conclusion:**

Social support plays a partial mediating role in the relationship between sleep quality and mental health among medical staff in township health centers.

## 1 Introduction

As an essential primary component of China’s healthcare service system, township health centers assume dual responsibilities of providing basic medical care and public health services. Their medical staff consistently work in environments characterized by heavy workloads, high occupational risks, and relatively limited resources. Persistent occupational stress may not only affect their job performance but also exert adverse effects on their physical and mental health (1, 2). Existing studies have indicated a high prevalence of sleep quality deterioration and mental health issues among medical staff (3–5), which may be particularly prominent in primary healthcare settings. Therefore, an in-depth exploration of the mechanism underlying the relationship between sleep quality and mental health among medical staff in township health centers holds significant practical importance.

From a theoretical perspective, this study is grounded in the stress-coping theory and the social support buffering hypothesis. The stress-coping theory suggests that when individuals face persistent stressors, their physiological and psychological systems generate stress responses. If coping resources are insufficient, this may lead to the development and exacerbation of mental health issues (6). Within this framework, sleep problems can be viewed both as manifestations of stress responses and as factors that further impair an individual’s coping capacity. Neuropsychological studies have found that diminished sleep quality impairs the functional connectivity of the prefrontal-limbic system, reducing emotional regulation capabilities and cognitive control levels (7, 8), thereby increasing the risk of psychological symptoms such as depression (9) and anxiety (10). Thus, sleep problems may directly impact mental health through the pathway of “sleep disruption → decreased emotional regulation capacity → increased psychological symptoms.”

Concurrently, the social support buffering hypothesis posits that social support, as a crucial external resource, can buffer the relationship between stress and health outcomes (11). Support from colleagues, family, and organizational levels can provide individuals with emotional support and problem-solving resources, enhancing their ability to cope with stress and thereby mitigating negative emotional responses and psychological symptoms. In the context of primary healthcare work, individuals with higher levels of social support may partially alleviate the psychological burden resulting from sleep problems through the mechanism of “social support → provision of emotional and coping resources → buffering stress effects.”

However, existing research has predominantly focused on medical staff in urban tertiary hospitals, with relatively limited attention paid to specific groups such as medical staff in township health centers. This group operates in environments with relatively scarce resources and potentially limited social support networks, and systematic empirical studies on the relationship between their sleep quality and mental health, as well as the underlying mechanisms, are still lacking. Therefore, it is necessary to conduct targeted research on this population guided by theoretical frameworks.

Based on the above theoretical analysis and current research status, this study proposes the following hypotheses: H1: Sleep quality is significantly correlated with mental health. H2: Sleep quality is significantly correlated with social support. H3: Social support plays a mediating role between sleep quality and mental health.

Thus, this study aims to investigate the relationship between sleep quality and mental health among medical staff in township health centers, with a specific focus on examining the mediating mechanism of social support in this relationship. The findings may provide a theoretical basis for developing mental health promotion strategies for primary healthcare workers and contribute to improving multi-level intervention systems, thereby fostering the stability and development of the primary healthcare workforce.

## 2 Materials and methods

### 2.1 Study participants

This study adopted a cross-sectional survey design and was conducted from September 1 to 20, 2024, in five districts and three counties of Lanzhou City, with medical staff from township health centers in these areas serving as the research subjects. A stratified random cluster sampling method was employed. Stratification was based on administrative divisions, dividing the five districts and three counties of Lanzhou into eight strata. Within each stratum, township health centers were treated as cluster units, and all township health centers within the jurisdiction were included for cluster investigation to achieve full regional coverage. In each included township health center, all medical staff meeting the inclusion criteria were enrolled as clusters. The sample covered all township health centers in the five districts and three counties of Lanzhou City, demonstrating good regional representativeness. This sampling approach helps reduce selection bias while ensuring regional representativeness, thereby enhancing the external applicability of the study findings.

### 2.2 Inclusion and exclusion criteria

Inclusion criteria: (1) Provided informed consent and voluntarily participated; (2) work experience ≥1 year and currently employed; (3) no severe organic diseases.

Exclusion criteria: (1) Individuals with a prior diagnosis of mental disorders, including those currently receiving psychiatric treatment or with a clear historical diagnosis; (2) non-clinical frontline staff.

The primary purpose of establishing these exclusion criteria is to prevent severe mental illnesses from interfering with the measurement outcomes of general mental health. This study aims to explore the relationship between sleep quality and general mental health levels, rather than conducting an epidemiological investigation of severe mental disorders. Therefore, excluding individuals with pre-existing mental disorders helps enhance sample homogeneity and improves the internal validity of the analysis of variable relationships.

Additionally, a supplementary explanation is provided in the discussion section: Due to the exclusion of individuals with previously diagnosed mental disorders, the observed incidence of psychological issues in this study may be lower than the actual prevalence rate within this population. Thus, the findings of this study are more applicable for assessing general mental health risk levels rather than estimating the overall prevalence of mental disorders.

### 2.3 Research tools

This study employed a questionnaire survey method, collecting data through a structured electronic questionnaire. The questionnaire consisted of the following four parts:

(1) Basic Information Questionnaire: Collected information on the subjects’ gender, age, ethnicity, occupation, educational level, work experience, and technical title. Educational level was categorized into four tiers: secondary specialized school, junior college, undergraduate, and postgraduate or above. “Secondary specialized school” refers to secondary vocational education, “junior college” refers to higher specialized education, “undergraduate” refers to standard higher education at the bachelor’s level, and “postgraduate or above” refers to master’s degree or higher.
(2) Pittsburgh Sleep Quality Index (PSQI): Used to assess the sleep quality of the subjects over the past month (12). The scale consists of 19 items divided into 7 dimensions, with a total score range of 0–21. According to common criteria, a total score >5 indicates poor sleep quality (13). In this study, the Cronbach’s *α* coefficient for this scale was 0.907.
(3) Symptom Checklist-90 (SCL-90): Used to evaluate the mental health status of the subjects over the past week. The scale contains 90 items divided into 10 factors (14). The total score ranges from 90 to 450, with a total score ≥150 indicating abnormal mental health status. In this study, the Cronbach’s *α* coefficient for this scale was 0.993.
(4) Social Support Rating Scale (SSRS): Used to assess the level of social support received by the subjects (15). It consists of 10 items divided into 3 dimensions. The total score ranges from 12 to 66, where a score ≤22 indicates low social support, 23–44 indicates medium social support, and ≥44 indicates high social support (16). In this study, the Cronbach’s *α* coefficient for this scale was 0.802.

### 2.4 Survey procedure

The electronic questionnaire was developed and distributed via the “Questionnaire Star” professional platform (V8.37). All investigators received unified training to ensure consistent understanding of the items.

A pilot survey was conducted prior to the formal study to clarify ambiguous expressions. Questionnaire links were distributed online to target healthcare workers. A total of 754 questionnaires were returned, with 745 valid responses, yielding an effective response rate of 98.75%.

### 2.5 Quality control and common method bias test

To ensure data quality, the first page of the questionnaire included detailed instructions and an informed consent procedure. Harman’s single-factor test indicated that the first common factor extracted without rotation explained 30.5% of the variance, which is below the critical threshold of 40%, suggesting no significant common method bias in the data. The study protocol was approved by the Medical Ethics Committee of Lanzhou Pulmonary Hospital (Approval No.: 202311101), and the rights and privacy of all participants were protected.

### 2.6 Statistical analysis

After exporting data from the Questionnaire Star platform and completing preliminary organization, statistical analysis was performed using SPSS 27.0 and AMOS 26.0 software. Normality tests showed that the measurement data did not follow a normal distribution; thus, they were described using the median and interquartile range [M (P25, P75)]. Categorical data were expressed as frequency and percentage [*n* (%)]. Cronbach’s *α* coefficient was used to evaluate the internal consistency reliability of the scales, and confirmatory factor analysis (CFA) was conducted to assess the structural validity of the measurement model. On the basis of controlling for demographic variables, partial correlation analysis was employed to explore the relationships among sleep quality, social support, and mental health. Furthermore, structural equation modeling (SEM) was constructed to examine the mediating role of social support between sleep quality and mental health. The mediating effect was tested using the Bootstrap resampling method (with 5,000 repeated samples) to estimate the standardized indirect effect and its 95% bias-corrected confidence interval (95% CI). A mediation effect was considered statistically significant if the confidence interval did not include zero. All statistical tests were two-tailed, and a *p*-value < 0.05 was considered statistically significant.

## 3 Results

### 3.1 Basic characteristics

As shown in Table 1, the majority of participants were female, of Han ethnicity, and nurses. Educational attainment was mainly at the junior college and bachelor’s degree levels. Working years were concentrated in the middle-aged and young backbone stage, with a distribution trend of high in the middle and low at both ends.

**Table 1:** Basic characteristics of surveyed township healthcare workers in Lanzhou City, 2023 [*n* (%)].

Characteristics	Number of participants (%)
Gender	
Female	672 (90.20)
Male	73 (9.80)
Age (years)	
≤25	74 (9.93)
26 ~ 35	372 (49.9 3)
36 ~ 45	233 (31.28)
≥46	66 (8.86)
Ethnicity	
Han	727 (97.58)
Ethnic minorities	18 (2.42)
Occupation	
Physicians	133 (17.85)
Nurses	612 (82.15)
Education	
Secondary specialized school	31 (4.16)
College	342 (45.91)
Bachelor’s degree	370 (49.66)
Master’s degree or above	2 (0.27)
Technical title	
None	246 (33.02)
Junior	301 (40.40)
Intermediate	170 (22.82)
Associate senior or above	28 (3.76)
Years of service	301 (40.40)
≤5	225 (30.20)
6 ~ 10	213 (28.59)
11 ~ 19	230 (30.87)
≥20	77 (10.34)

### 3.2 Current status of sleep quality, social support, and mental health

Among the surveyed township healthcare workers, 415 individuals had sleep quality problems, yielding a sleep disorder detection rate of 55.70%. The SCL-90 assessment identified 339 individuals with positive psychological symptoms, yielding a positive rate of 45.50%. As shown in Table 2, the level of social support among the participants was moderate.

**Table 2:** Scores of scales and dimensions among surveyed township healthcare workers in Lanzhou City, 2024 [M (P25, P75)].

Dimension	Score
SCL-90 total	137.00 (101.00, 183.50)
Somatization	19.00 (13.50, 25.00)
Obsessive-compulsive	19.00 (13.00, 23.00)
Interpersonal sensitivity	14.00 (10.00, 19.00)
Depression	20.00 (14.00, 27.50)
Anxiety	14.00 (11.00, 20.00)
Hostility	9.00 (7.00, 12.00)
Phobic anxiety	9.00 (7.00, 14.00)
Paranoid ideation	8.00 (6.00, 12.00)
Psychoticism	13.00 (10.00, 19.00)
Other	11.00 (7.00, 15.00)
PSQI total	6.00 (4.00, 9.00)
Sleep duration	1.00 (0.00, 2.00)
Sleep efficiency	0.00 (0.00, 1.00)
Sleep latency	1.00 (1.00, 2.00)
Sleep disturbance	1.00 (1.00, 1.00)
Subjective sleep quality	1.00 (0.00, 2.00)
Use of sleep medication	0.00 (0.00, 0.00)
Daytime dysfunction	1.00 (1.00, 2.00)
SSRS total	38.00 (32.00, 43.00)
Objective support	7.00 (6.00, 8.00)
Subjective support	24.00 (19.00, 27.00)
Support utilization	7.00 (6.00, 9.00)

### 3.3 Correlation analysis among sleep quality, social support, and mental health

As shown in Table 3, poorer sleep quality was associated with more severe mental health problems (*r* = 0.602, *p* < 0.001) and lower social support levels (*r* = −0.272, *p* < 0.001). Higher social support was associated with better mental health (*r* = −0.305, *p* < 0.001).

**Table 3:** Correlation analysis among PSQI, SCL-90, and SSRS scores among surveyed township healthcare workers in Lanzhou City, 2024 (*r*).

Variable	PSQI total score	SSRS total score	SCL-90 total score
PSQI total score	1.000***		
SSRS total score	−0.272***	1.000***	
SCL-90 total score	0.602***	−0.305***	1.000***

***indicates *p* < 0.001.

### 3.4 Path analysis of the effects of sleep quality and social support on mental health

Limitations Section: Although this study provided a theoretical explanation for model modification, the specification of residual correlations was not pre-hypothesized but rather a posteriori adjustment based on model fitting. Therefore, this modification may be influenced to some extent by sample-specific characteristics and requires confirmation in future studies through cross-validation or multi-sample comparisons.

Model Fitting Section: To test the theoretical hypothesis model, structural equation modeling (SEM) was constructed and fitted using AMOS 27.0 software. The initial model showed no improper estimates (e.g., negative variances or excessively large standard errors), but the overall model fit was suboptimal (*χ*^2^/df = 7.771, GFI = 0.832, RMSEA = 0.095, CFI = 0.937, TLI = 0.928, NFI = 0.929, PNFI = 0.809, PCFI = 0.817). According to the Modification Index (MI) output by AMOS, the residual covariance between the two observed variables of anxiety and depression had a high modification index, indicating shared variance not explained by the model.

From a theoretical perspective, anxiety and depression symptoms exhibit high overlap in the dimension of negative affect. Factor structure analyses of standard scales suggest that they share substantial common variance and reflect the latent construct of negative emotion rather than fully independent dimensions (17, 18). Neuroimaging studies have shown that patients with depression and anxiety disorders exhibit prefrontal dysfunction and impaired connectivity with the limbic system during facial emotion processing and automatic emotion regulation tasks, suggesting that prefrontal-limbic dysfunction may be the neural basis for the comorbidity of these two emotional symptoms (19). Therefore, allowing the residuals of anxiety and depression to correlate in the model is theoretically justifiable and not merely a data-driven statistical adjustment.

Statistically, only this specific residual covariance was modified in the model, avoiding multiple data-driven path adjustments to prevent overfitting. The modified model showed significantly improved fit indices (*χ*^2^/df = 4.899, GFI = 0.900, RMSEA = 0.072, CFI = 0.966, TLI = 0.959, NFI = 0.957, PNFI = 0.795, PCFI = 0.802), all of which met recommended standards, indicating good model-data fit suitable for subsequent path analysis.

In the structural equation modeling analysis, after model fitting and necessary modifications, a final mediation structural model incorporating sleep quality, social support, and mental health was established. As shown in Table 4, all path coefficients were statistically significant (*p* < 0.01). The standardized path coefficients among the latent variables in the structural model ranged from −0.304 to 0.627, indicating moderate correlations among the variables.

**Table 4:** Path coefficients of the structural model.

Path	Std	Unstd	SE	*t*-value	*p*-value
PSQI → SSRS	−0.304	−0.401	0.068	−5.934	<0.01
SSRS → SCL-90	−0.164	−1.458	0.331	−4.409	<0.01
PSQI → SCL-90	0.627	7.349	0.488	15.069	<0.01

As presented in Table 5, the standardized factor loadings of the observed variables on their corresponding latent variables in the measurement model ranged from 0.246 to 0.975, with most factor loadings reaching or approaching 0.50 or above, suggesting acceptable measurement validity of the overall model.

**Table 5:** Factor loading coefficients of the measurement model.

Path	Std	Unstd	SE	*t*-value	*p-*value	SMC	CR	AVE
PSQI → daytime dysfunction	0.744	1.000			<0.01	0.554	0.772	0.348
PSQI → use of sleep medications	0.395	0.343	0.036	9.596	<0.01	0.156		
PSQI → sleep disturbances	0.748	0.762	0.041	18.542	<0.01	0.560		
PSQI → sleep efficiency	0.246	0.284	0.047	6.107	<0.01	0.061		
PSQI → sleep duration	0.454	0.549	0.049	11.325	<0.01	0.206		
PSQI → sleep latency	0.621	0.796	0.052	15.218	<0.01	0.386		
PSQI → subjective sleep quality	0.718	0.874	0.048	18.085	<0.01	0.516		
SSRS → objective support	0.641	1.000			<0.01	0.411	0.682	0.417
SSRS → subjective support	0.664	3.898	0.341	11.440	<0.01	0.441		
SSRS → utilization of support	0.632	1.570	0.146	10.782	<0.01	0.399		
SCL-90 → somatization	0.933	1.000			<0.01	0.870	0.989	0.912
SCL-90 → obsessive-compulsive symptoms	0.947	0.886	0.017	53.152	<0.01	0.897		
SCL-90 → interpersonal sensitivity	0.966	0.800	0.015	52.615	<0.01	0.933		
SCL-90 → depression	0.967	1.179	0.020	58.455	<0.01	0.935		
SCL-90 → anxiety	0.975	0.865	0.014	61.024	<0.01	0.951		
SCL-90 → hostility	0.946	0.530	0.010	52.959	<0.01	0.895		
SCL-90 → phobic anxiety	0.945	0.584	0.011	52.480	<0.01	0.893		
SCL-90 → paranoid ideation	0.957	0.511	0.009	55.593	<0.01	0.916		
SCL-90 → psychoticism	0.960	0.833	0.015	56.142	<0.01	0.922		

In terms of structural path relationships, sleep quality significantly and negatively predicted social support (*β* = −0.304, *p* < 0.01) and significantly and positively predicted mental health (*β* = 0.627, *p* < 0.01). Social support had a significant positive effect on mental health (*p* < 0.01) and played a significant mediating role between sleep quality and mental health. The results indicate that sleep quality not only directly affects mental health but also exerts an indirect effect through the mediating variable of social support, demonstrating a partial mediation pattern.

Regarding reliability and validity testing, the squared multiple correlations (SMC) of the measurement indicators for the sleep quality dimension ranged from 0.061 to 0.560. The composite reliability (CR) was 0.772, meeting the recommended criterion of above 0.70. However, the average variance extracted (AVE) was 0.348, which is below the ideal threshold of 0.50, indicating some limitations in the convergent validity of this latent variable. For the social support dimension, the SMC values ranged from 0.399 to 0.441. The CR was 0.682, slightly below the recommended standard of 0.70, and the AVE was 0.417, also falling short of the ideal level of 0.50. This suggests that the internal consistency and convergent validity of this dimension require further optimization. For the mental health dimension, the SMC values ranged from 0.870 to 0.951. The CR was 0.989, and the AVE was 0.912, both far exceeding the recommended standards, indicating good internal consistency and convergent validity for this latent variable.

Although the AVE of some latent variables did not reach the ideal standard of 0.50, their CR values generally met or approached 0.70. According to the criteria proposed by Fornell and Larcker (20), if the CR exceeds 0.60, an AVE slightly below 0.50 can still be considered indicative of acceptable convergent validity. Therefore, the overall reliability and validity of the measurement model in this study are within an acceptable range. However, future research could further enhance model quality by optimizing measurement items or removing low-loading indicators.

After controlling for demographic and occupation-related variables, the mediation model results indicated that the total effect of sleep quality on mental health was 7.933 (95% CI: 6.697–9.354, *p* = 0.009). Among this, the direct effect was 7.349 (95% CI: 6.194–8.722, *p* = 0.008), accounting for 92.64% of the total effect. The indirect effect was 0.584 (95% CI: 0.492–0.671, *p* = 0.002), accounting for 7.36% of the total effect. Bootstrap testing showed that the confidence interval of the indirect effect did not include zero, indicating that social support played a significant partial mediating role between sleep quality and mental health. These results suggest that the impact of sleep quality on mental health is primarily realized through the direct pathway, while a smaller portion of the effect is transmitted indirectly through the mediating variable of social support. The relatively low proportion of the indirect effect implies that although social support demonstrates a statistically significant mediating role, its practical contribution to the overall effect is limited. Possible reasons for this phenomenon include: The mental health of medical staff may be more directly influenced by individual physiological mechanisms; Social support, as a psychosocial variable, operates through a relatively indirect pathway; The mediating effect, while statistically significant, is small in magnitude, representing a case of “statistically significant but practically limited” mediation.

## 4 Discussion

This study revealed that the detection rate of sleep disorders (55.70%) and the positive rate of mental health issues (45.50%) among medical staff in township health centers were both at relatively high levels, indicating that primary healthcare practitioners may face prominent physical and mental health risks. Sleep quality was significantly positively correlated with mental health, while social support was significantly negatively correlated with both. The overall results are consistent with existing theoretical expectations. It is important to emphasize that, given this study’s cross-sectional design, the relationships between these variables should be interpreted as statistical associations rather than causal inferences. Compared with previous studies (21–23), the levels of sleep disorders and positive mental health screenings in this study fall within the medium-to-high range. These differences may be related to the occupational characteristics of the sample. Medical staff in township health centers work long-term in environments with relatively limited human resources and overlapping multiple tasks. Unstable work rhythms and persistent occupational stress are likely associated with their sleep and emotional states. This finding, to some extent, supports the “high-demand and insufficient recovery” framework within occupational stress models, suggesting that restricted physiological recovery may be linked to increased levels of psychological exhaustion (24).

Correlation analysis further indicated that higher sleep quality scores were significantly associated with elevated mental health risk levels, whereas higher social support levels were correlated with lower levels of sleep problems and psychological risks. These results align with the stress-coping theory and the social support buffering hypothesis, suggesting that social support may serve as an important psychosocial resource closely related to individual stress experience and emotional state.

The structural equation modeling results demonstrated a significant direct path between sleep quality and mental health, while social support played a significant partial mediating role between them. However, the mediation effect accounted for only 7.36% of the total effect, indicating that the association between sleep quality and mental health is primarily manifested through direct pathways. This finding possesses certain mechanistic plausibility. Firstly, from the physiological-psychological pathway perspective, sleep disturbances may be linked to anxiety and depressive symptoms through mechanisms such as disrupting hypothalamic–pituitary–adrenal (HPA) axis function, activating inflammatory response pathways, and affecting neurotransmitter regulation processes. Studies have indicated that abnormal HPA axis rhythms are closely associated with sleep disorders, while elevated inflammatory markers like IL-6 and TNF-α may mediate the connection between sleep deprivation and mood disorders (25). Furthermore, neurobiological research on insomnia suggests that sleep problems may impact neurotransmitter systems such as 5-HT and GABA, thereby interfering with emotional regulation mechanisms (26). Therefore, a relatively direct biological pathway may exist between sleep quality and mental health, resulting in the dominance of the direct effect.

Social support, as a psychosocial resource, has been demonstrated in numerous studies to buffer stress and promote psychological adaptation. According to the Social Support Buffering Hypothesis, social support can reduce subjective stress perception and enhance an individual’s coping capacity, thereby correlating with better mental health (27). Furthermore, Relationship Regulation Theory posits that social support influences emotional regulation processes through daily relational interactions, which are closely associated with subjective mental health levels (28). Relevant reviews also indicate that by improving emotional regulation ability and psychological resilience, social support helps mitigate negative emotional experiences (29).

Therefore, in populations where physiological fatigue and occupational load are the primary stressors, the proportional mediating effect of social support may be relatively limited. Nonetheless, its statistical significance suggests a stable role within the psychological adaptation system. Additionally, as the sample consists of primary healthcare workers, the psychological state of this group may be more strongly linked to insufficient physiological recovery and persistent occupational stress. Within this context, the association between sleep quality—a key indicator of recovery mechanisms—and mental health may be more direct, while social support likely plays a supplementary buffering role.

The theoretical significance of this study lies in incorporating both sleep quality and social support within a unified structural model framework, validating their interrelationships in the context of primary care settings, and providing empirical support for understanding the multi-pathway mechanisms influencing the mental health of occupational groups. The results suggest that sleep problems may be a significant correlational factor affecting mental health, while social support, as a psychosocial resource, plays a certain yet limited mediating role in this relationship.

From the perspective of organizational-individual interaction mechanisms, the mental health issues among medical staff in primary care institutions are not merely individual-level stress responses but rather outcomes resulting from the combined effects of work structure characteristics and resource allocation conditions. Occupational Stress Models and the Job Demands-Resources (JD-R) Model indicate that when high workload persists alongside insufficient recovery resources, individuals are more prone to psychological exhaustion and impaired emotional functioning. Therefore, within the primary healthcare system context, optimizing scheduling structures and ensuring adequate recovery time after continuous work can help reduce sustained physiological arousal levels, thereby buffering the cumulative effects of stress at its source. Meanwhile, organizational-level psychological support mechanisms—such as mental health liaisons and peer support groups—can be regarded as extended forms of “job resources.” These help enhance emotional regulation capacity and coping efficacy, thereby reducing the risk of occupational burnout and psychological distress (30). At the county-township collaboration level, initiatives such as touring guidance (circuit guidance) by professionals, job rotation exchanges, and the establishment of remote psychological support systems can structurally improve primary care workers’ access to professional support. Such institutional support not only increases the supply of objective resources but also indirectly enhances psychological resilience and emotional regulation ability by strengthening perceived support and career development expectations (31). From a longer-term perspective, integrating mental health monitoring into routine management indicators and improving mechanisms for replenishing the primary care workforce can help build a stable supportive environment at the organizational level, mitigating the erosion of mental health by chronic stress exposure. Additionally, family-involved support programs can expand the external structure of individuals’ social support networks, buffering the adverse effects of stress on emotional states by enhancing co-regulatory processes within close relationships. Thus, within the primary healthcare system, intervention strategies should be advanced synergistically at two levels: “strengthening individual coping capacity” and “redistributing organizational resources.” By improving recovery conditions, enhancing social support, and increasing professional development opportunities, a multi-level psychological protection network can be constructed to mechanistically reduce the adverse impact of sustained high-workload environments on mental health (Table 6).

**Table 6:** Effect decomposition of the mediation model (standardized).

Effect type	Effect value	95%CI	*p*-value	Proportion (%)
Total effect	7.933	6.697 ~ 9.354	0.009	
Direct effect	7.349	6.194 ~ 8.722	0.008	92.64
Indirect effect	0.584	0.492 ~ 0.671	0.002	7.36

This study also has several limitations. The cross-sectional design restricts causal inference; the imbalanced gender distribution in the sample may affect the generalizability of the results; and due to the exclusion of individuals with pre-existing diagnosed mental disorders, the reported prevalence of psychological issues in this study may be lower than the true prevalence rate in this population. Therefore, the findings of this study are more applicable for assessing general mental health risk levels rather than the overall prevalence of mental disorders. Additionally, although the model modifications were theoretically justified, they may still be sample-specific. Future studies could employ longitudinal designs or multi-sample validation to further examine the model’s stability and explore other potential mediating mechanisms—such as emotional regulation capacity, occupational burnout, or coping styles—to more comprehensively elucidate the pathways linking sleep and mental health.

## Data Availability

The original contributions presented in the study are included in the article/supplementary material, further inquiries can be directed to the corresponding authors.
